# Age-Related Dysfunction in Balance: A Comprehensive Review of Causes, Consequences, and Interventions

**DOI:** 10.14336/AD.2024.0124-1

**Published:** 2024-01-24

**Authors:** Jixian Wang, Yongfang Li, Guo-Yuan Yang, Kunlin Jin

**Affiliations:** ^1^Department of Rehabilitation Medicine, Ruijin Hospital, Shanghai Jiao Tong University School of Medicine, Shanghai, China.; ^2^Neuroscience and Neuroengineering Research Center, Med-X Research Institute and School of Biomedical Engineering, Shanghai Jiao Tong University, Shanghai, China.; ^3^Department of Pharmacology and Neuroscience, University of North Texas Health Science Center, Fort Worth, TX 76107, USA

**Keywords:** aging, balance, fall, rehabilitation, therapy

## Abstract

This review delves into the multifaceted aspects of age-related balance changes, highlighting their prevalence, underlying causes, and the impact they have on the elderly population. Central to this discussion is the exploration of various physiological changes that occur with aging, such as alterations in the vestibular, visual, proprioceptive systems, and musculoskeletal degeneration. We examine the role of neurological disorders, cognitive decline, and medication side effects in exacerbating balance issues. The review underscores the significance of early detection and effective intervention strategies in mitigating the risks associated with balance problems, such as falls and reduced mobility. It discusses the effectiveness of diverse intervention strategies, including exercise programs, rehabilitation techniques, and technological advancements like virtual reality, wearable devices, and telemedicine. Additionally, the review stresses the importance of a holistic approach in managing balance disorders, encompassing medication review, addressing comorbidities, and environmental modifications. The paper also presents future research directions, emphasizing the need for a deeper understanding of the complex mechanisms underlying balance changes with aging and the potential of emerging technologies and interdisciplinary approaches in enhancing assessment and intervention methods. This comprehensive review aims to provide valuable insights for healthcare providers, researchers, and policymakers in developing targeted strategies to improve the quality of life and ensure the well-being of the aging population.

## 1. Overview

### Aging, Balance Disorders, and the Growing Need for Effective Interventions

Aging and the diseases caused by aging are among the most insurmountable social and medical problems worldwide [1-3]. The aging and aging related diseases such as stroke, traumatic brain injury (TBI), and Parkinson’s disease (PD) are closely related to a variety of physiological changes that affect motor, sensory, and cognitive functions [4, 5]. There is growing evidence that balance disturbance is the main symptom of aging in older adults [6]. Therefore, understanding the mechanisms of balance disorders and developing effective early detection and intervention becomes crucial. Falls are a great cost to society. Approximately $50 billion is spent on non-fatal falls and another $754 million on 28,486 fatal falls yearly [7]. Most falls happen in elderly populations, and even more in populations with balance deficits associated with stroke, TBI, or PD [8, 9]. The prevalence of balance disorders in the elderly population is relatively high and increases with age. About 30% of adults aged 65 and over will experience balance problems or dizziness at some point in their lives [10, 11].

In addition, people over the age of 75 tend to have higher rates of balance disorders. The most common balance disorders in the elderly population include age-related decreased balance and postural stability, benign paroxysmal positional vertigo (BPPV), vestibular neuritis, and Meniere's disease [12]. These conditions could seriously affect older adults' ability to perform daily activities, increase the risk of falls, and lead to loss of motor independence [13]. Balance disorders in older adults could be influenced by multiple factors, including age-related changes in sensory systems such as vision, vestibular, and somatosensory, musculoskeletal loss, changes in joint mobility and bone density, cognitive factors, and underlying medical conditions [14, 15]. The study of relationships between aging and balance is a multidisciplinary field involving biology, medicine, physiology, psychology, and biomechanics [16]. Over the years, researchers have made considerable progress in understanding the changes in balance and stability that occur as individuals age.

Throughout history, there have been anecdotal observations of balance difficulties in older adults [17]. These observations often attribute balance problems to the general weakness and frailty associated with aging. However, systematic scientific research on balance and its changes with age has only recently begun. In the early 20th century, the field of gerontology began to focus on the study of aging and its effects, including balance and mobility [18]. Early studies often relied on clinical observations and basic physiological assessments to understand age-related changes in balance. In the mid-20^th^ century, researchers began to develop sophisticated balance assessment tools and methods [19]. These include balance boards, force plates, and other equipment that can measure postural stability. These advances allow for more quantitative and objective measurements of balance, leading to a better understanding of specific changes that occur with aging [20]. In the late 20^th^ century, as technology continued to advance, researchers gained a better understanding of the biomechanical and neurological aspects of balance control [21]. Research is beginning to focus on the role of senses such as vision, proprioception, and the vestibular system in maintaining balance, and how these sensory systems change with age [22]. Researchers have also explored how muscle strength, joint function, and cognitive factors influence balance in older adults [23-25]. Since the beginning of the 21^st^ century, the study of aging and balance has increasingly become an interdisciplinary study involving researchers in the fields of medicine, biomechanics, neuroscience, psychology, and physical therapy [26, 27]. As the aging population grows, greater emphasis is placed on understanding balance deficits and developing interventions to improve balance and prevent falls in older adults. Research has explored interventions such as exercise programs, balance training and rehabilitation techniques to enhance balance and reduce the risk of falls in older adults [28, 29]. 21^st^ century technology has also advanced, allowing for more sophisticated and non-invasive assessments of balance and stability. Researchers are investigating the role of genetics, cellular and molecular mechanisms, and neuroplasticity in age-related changes in balance [30-32]. Virtual reality and wearable devices are already being used to create immersive training environments to improve balance in older adults [33, 34]. Overall, the study of aging and balance has grown from anecdotal observations into a well-established field, with a greater understanding of the physiological, biomechanical, and neural factors that lead to changes in balance in older adults. Ongoing research continues to refine our knowledge and inform interventions to promote healthy aging and reduce falls-related risk.

### 1.2. Advancements in Balance Disorders and Aging Research

In 1893, French otologist Prosper Ménière published a paper entitled "Sur une forme de surditégrave dépendant d'une lésion de l'oreille interne*"* [35]. In that paper, Meniere attributed the characteristic symptoms of clinical trials to vertigo, fluctuating hearing loss and tinnitus, and pointed out that these symptoms were related to inner ear diseases, later identified as 'Ménière's disease' by scientists”. Since then, many advances have been made in the understanding and classification of balance disorders [36]. Balance disorders have since been recognized as distinct medical conditions. The field of balance disorders research has evolved significantly since Menier's original contributions.

The importance of balance problems in older adults has been recognized for centuries, but early explanations often attributed these symptoms to age-related decline or frailty. Ancient medical texts from various cultures occasionally refer to balance issues in the elderly, but scientific understanding of the mechanisms is limited. Epidemiological studies in the middle and late 20^th^ century began to study the prevalence and risk factors of balance disorders in the elderly [37]. Scientists are beginning to explore age-related changes in the vestibular system, which is critical for balance and spatial orientation. Studies found that certain vestibular functions, such as vestibulo-ocular reflex (VOR) gain, may decline with age, leading to balance problems in older adults [38, 39]. Falls are an important problem in the elderly population, and balance impairment plays a key role in the risk of falls. The prevention of falls in older adults has led to increased awareness of the importance of balance training and rehabilitation interventions. Physiotherapists and geriatricians develop specialized rehabilitation programs to address balance disorders in older adults. For example, vestibular rehabilitation therapy (VRT) is specifically designed to meet the unique needs of older adults [40].

In addition, age-related changes in multisensory integration, such as vision, proprioception, and vestibular function, can also affect the ability to maintain balance. Advances in diagnostic tools, including imaging technologies and wearable sensors, allow for more detailed assessments of balance function in the aging population [41]. It is worth noting that treatment without evaluation is ineffective. Furthermore, studying balance disorders in older adults requires an interdisciplinary approach involving clinicians and scientists from multiple fields, including geriatrics, otology, neurology, physical therapy, and epidemiology. Collaboration between these disciplines leads to a more comprehensive understanding of the balance of population ageing. Longitudinal studies have played a significant role in tracking changes in balance in older adults over time.

Given that the world's population is aging rapidly, with an estimated 2.1 billion people aged 60 and over globally by 2050, understanding age-related shifts in balance is critical to addressing the health and well-being of older adults [41]. Studying age-related changes in balance is critical to addressing the challenges posed by an aging population, preventing falls, reducing healthcare burden, and promoting healthy aging and functional independence. Findings from this study could inform evidence-based interventions and policies aimed at improving the well-being and quality of life of older people around the world.

### 1.3. Purpose of this review

This review will provide key findings, research methods and gaps in existing knowledge on age-related changes in balance. Physiological, sensory, neurological, cognitive, and musculoskeletal factors contributing to age-related changes in balance will be analyzed. Simultaneously identify modifiable and non-modifiable risk factors associated with age-related balance changes and fall risk to inform targeted interventions and fall prevention strategies. We evaluate the effectiveness of various interventions aimed at improving balance in older adults, discuss the impact of age-related changes in balance on fall prevention efforts and the importance of early detection and intervention. Finally, we will address the limitations of the current research and suggest areas for further research, including the integration of new technologies, interdisciplinary collaborations, and longitudinal studies. Provides valuable insights for healthcare providers and policy makers to develop targeted healthcare strategies that address age-related shifts in balance and support healthy aging.

## 2. Age-related changes in neurological sensory systems

### 2.1. Age-related changes in sensory systems

#### 2.1.1 Effects of visual changes on balance

The visual system plays a vital role in maintaining balance and spatial orientation [42]. It provides important cues about the position and movement of the body relative to the environment. When visual input is impaired, it can lead to balance problems and increase the risk of falls, especially in older adults. Visual changes that affect balance may be due to reduced depth perception, altered field of view, decreased vision, decreased sensitivity to light, and impaired visual tracking [43]. For the elderly, it is difficult to accurately judge the distance between objects and themselves [44]. It can lead to misjudging steps or uneven surfaces, increasing the risk of tripping and falling. Visual acuity refers to the clarity of vision, which gradually decreases with age. Poor vision could make it difficult to recognize obstacles, curbs, or spot hazards, which could lead to balance problems.

Some older adults experience an increased sensitivity to bright light or glare as they age. This sensitivity may affect the ability to see clearly in certain environments, thereby affecting balance and stability. Impaired visual tracking due to changes in vision or certain neurological disorders could affect a person's balance, especially during activities that involve head or eye movement [45]. Transitioning between different lighting conditions such as moving from a bright room to a dark hallway could pose challenges for individuals with visual changes and increased risk of falls [46]. This difficulty adjusting to changing light levels can cause temporary disorientation and increase the risk of falls.

#### 2.1.2 The effect of the vestibular system on balance

The vestibular system plays a vital role in maintaining balance and orientation in space. It is the complex sensory system located in the inner ear that detects head movement and changes in head position relative to gravity. This information is critical for coordinating body movements and stabilizing sight during head movements. The main components of the vestibular system are the three fluid-filled semicircular canals (horizontal, anterior, and posterior ear canals), the otoliths, and the vestibular nerve pathway that connects the brainstem to the cerebellum. When we turn our head in any direction, the fluid in these ear canals moves, stimulating the hair cells that transmit signals to the brain about the direction and speed of head movement [47]. The otoliths are responsible for detecting the effects of linear acceleration and gravity and could respond to changes in head position with respect to gravity and linear motion.

The vestibular system works closely with the visual system to stabilize gaze during head movements. It helps to focus the eyes on the target during head movement to see clearly and maintain vision. The vestibular system provides constant feedback to the brain about the body's position and movement in space, and the vestibular system plays a vital role in a variety of everyday activities such as maintaining an upright posture, walking, running, and even more complex movements such as dancing and sports [48]. With age, certain changes occur in the vestibular system, including a decrease in the number of hair cells, slower reaction times, decreased otolith function, and impaired gaze stability [49]. These age-related changes in the vestibular system may increase the risk of balance problems, dizziness, and falls in older adults.

#### 2.1.3 The effect of proprioception on balance

Human beings could perceive their position, movement, and orientation in space, which is called proprioception. It involves sensors located in muscles, tendons and joints that provide the brain with constant feedback about the internal state of the body. Help the brain maintain body awareness and control movement without relying on visual input. Proprioceptive abilities decrease with age, which could have a major impact on balance. For example, the number and sensitivity of proprioceptive receptors in muscles, tendons, and joints decreases with age. This could lead to less accurate detection of joint angles and muscle lengths, affecting the brain's ability to precisely coordinate movements [50].

In addition, aging causes changes in the CNS that processes and integrates proprioceptive information. These neural changes slow the transmission of sensory signals, affecting the speed and accuracy of motor responses needed to maintain balance. Age-related loss of muscle mass and strength could also lead to decreased joint stability and control. Weaker muscles may have difficulty providing adequate support during weight-bearing activities, leading to balance problems [51]. Osteoarthritis and other joint-related conditions cause pain, stiffness, and reduced range of motion with age, affecting proprioceptive feedback and balance. Decreased proprioception can lead to balance disorders, especially in older adults [52]. Balance is a complex process that involves the integration of inputs from the vestibular, visual, and proprioceptive systems. When proprioceptive input is impaired, older adults may rely more on their visual and vestibular systems for balance [50]. Reduced proprioception may increase the risk of falls, leading to difficulty with gait, impaired joint stability, and slower reaction times.

### 2.2. The central effect of cerebellum on the balance control

Cerebellum plays a crucial role in motor control and coordination. While it is often associated with the coordination of voluntary movements, it also has a significant impact on balance and posture [53]. The cerebellum receives input from various sensory systems and proprioceptive information from muscles and joints. It integrates this information to create a comprehensive representation of the position of body and movement in space [54]. Impotently, the cerebellum acts as a feedback control system. It continuously receives sensory input regarding the body's position and movement and compares this information with the intended movement [55]. If there is a mismatch, the cerebellum sends corrective signals to adjust muscle activity, contributing to balance maintenance [56]. The cerebellum is involved in motor learning and adaptation and influences the timing and coordination of muscle contractions during walking (gait). It contributes to the rhythmic and coordinated movements involved in maintaining an upright posture during walking [57]. In response to changes in body position, the cerebellum contributes to the generation of appropriate postural adjustments [58]. This involves coordinating muscle activity to prevent falls and maintain stability.

Disruptions in the cerebellum could lead to balance problems and coordination difficulty. Head trauma could cause direct damage to the cerebellum, causing problems with balance, coordination, and fine motor movements. Certain neurodegenerative diseases such as multiple system atrophy, spinocerebellar ataxia, and Friedreich's ataxia, cause progressive dysfunction and degeneration of the cerebellum, which leads to movement abnormalities and other neurological deficits [59]. Clinical assessments of balance often involve observing a person's ability to stand, walk, and perform coordinated movements [60]. Balance exercises and rehabilitation are recommended to improve cerebellar function and address balance issues in individuals with neurological conditions affecting the cerebellum [61]. Neurodegenerative diseases that affect the cerebellum, such as spinocerebellar ataxia causes gait ataxia and balance problems. Individuals may exhibit a broad-based, unsteady gait and have difficulty maintaining balance while standing and walking [62].

### 2.3. Effects of neurotransmitters and balance-related brain parts on balance

Neurotransmitters play a vital role in maintaining the normal function of the vestibular system and are closely related to balance control in the elderly population [63]. The vestibular system relies on communication between nerve cells and the release of neurotransmitters to convey messages about balance and balance [64]. Several key neurotransmitters participate in vestibular system and balance control, including glutamate, GABA, acetylcholine, serotonin, and dopamine. The levels or functions of these neurotransmitters change with age and could contribute to balance disorders in the elderly population [65]. Age-related decline in the function of the vestibular system, changes in the neurotransmitter system, or changes in communication between nerve cells could lead to balance problems, dizziness, and a higher risk of falls in older adults.

The vestibular nucleus plays a crucial role in balance control, and its function becomes especially important in the context of balance disorders in the elderly [66]. The vestibular nucleus is a group of four neuronal clusters located in the brainstem, particularly in the medulla and pons. They receive input from the vestibular organs of the inner ear, which process and integrate sensory information related to head movements and changes in body position. In the elderly population, some changes could occur in the vestibular system and the vestibular nuclei such as the sensory hair cells in the vestibular organ could degenerate or become less sensitive to movement, and the degeneration of the vestibular nucleus neurons leads to a reduction in the number of functional neurons [67]. The vestibular nuclei are responsible for integrating sensory information from the vestibular organs with input from other sensory systems. With age, the ability of the CNS to integrate these sensory inputs could weaken, affecting balance and postural coordination. Blood flow to the brainstem and vestibular nuclei could be affected by age-related vascular changes, which further affect the function of these structures and lead to balance disturbances.

The integrity of white matter pathways plays a critical role in maintaining balance and motor function in aging populations. White matter in the brain is made up of myelinated nerve fibers that form neural pathways, allowing different regions of the brain to communicate and coordinate complex motor activity. These pathways are critical for the transfer of information between the cerebellum, brainstem, and various sensorimotor regions involved in balance control [68]. Age-related changes in white matter integrity affect the transmission of neural signals, resulting in impaired balance in older adults [69]. White matter integrity naturally reduces as individuals age. White matter microstructure, such as reduced myelin density and axonal damage, could disrupt the efficient transmission of signals between brain regions involved in balance and motor control. Reduced white matter integrity is associated with abnormal gait, increased risk of falls, and impaired balance in older adults [70]. White matter integrity is critical for cognitive-motor integration, and impairment of this integration could lead to balance disturbances and increase fall risk in older adults. Disruption of white matter pathways that connect different sensory and motor regions in the brain could lead to deficits in sensorimotor integration and postural adjustment.

The brain's vascular system plays a vital role in supplying oxygen and nutrients to areas of the brain involved in balance control. Any disruption of blood flow to these areas could cause balance problems. Transient ischemic attack, vertebrobasilar insufficiency, and stroke cause sudden and severe balance disturbances due to reduced blood supply to specific brain regions [71, 72]. As people age, decreased cerebral blood flow and perfusion, particularly in the cerebellum and brainstem, could lead to balance disorders. White matter hyperintensities are areas of increased signal intensity on MRI scans of the brain, and these hyperintensities are associated with balance impairment and an increased risk of falls in older adults [73]. With orthostatic hypotension, a sudden drop in blood pressure cause dizziness and balance problems, increasing fall risk. Atherosclerosis results in decreased blood flow, oxygen and nutrient supply to areas involved in balance control. Microvascular dysfunction involves damage to the small blood vessels in the brain, resulting in reduced blood flow to areas of balance control. Vascular dementia is a form of dementia caused by decreased blood flow to the brain, which induced cognitive impairment and caused balance and gait disturbances.

### 2.4. Effects of neurodegeneration on balance

Neurodegenerative diseases are a group of disorders characterized by the progressive degeneration of nerve cells in the brain and CNS. Neurodegenerative diseases affect various areas of the brain that are critical for the coordination and regulation of balance, leading to balance impairment and increased fall risk. Parkinson's disease (PD), Alzheimer's disease (AD), multiple system atrophy (MSA), Huntington's disease (HD), multiple sclerosis, amyotrophic lateral sclerosis (ALS), corticobasal degeneration (CBD), progressive nuclear superior paresis (PSP) and dementia with Lewy bodies are the most common neurodegenerative disorders affecting balance control [74]. PD primarily involves the degeneration of dopamine-producing neurons in the basal ganglia. These results in a stooped posture, decreased arm swing, and difficulty starting or stopping movements. AD causes progressive cognitive reduction, memory loss and impaired judgment. These cognitive impairments affect the ability to process sensory information, make balance-related decision, and recognize environmental hazards, leading to an increased fall risk [75]. Some neurodegenerative diseases could cause peripheral neuropathy, resulting in sensory deficits, weakness, and loss of proprioception, all of which affect balance and stability [76]. Autonomic nervous system dysfunction occurs in certain neurodegenerative diseases. Autonomic dysfunction led to orthostatic hypotension, dizziness, and balance problems. In all these neurodegenerative diseases, the degeneration of specific brain regions or pathways disrupts the complex neural networks responsible for maintaining balance and posture. The resulting balance impairment can severely impair mobility, increase the risk of falls, and reduce overall quality of life [77].

### 2.5. Impact of cognitive reduction on balance disorders

Cognitive decline involves a gradual deterioration in cognitive abilities such as memory, attention, problem-solving, and decision-making, which is associated with balance disorders [78]. Cognitive functions play a crucial role in maintaining balance and stability as they involve processing sensory information, coordinating movements, and making real-time adjustments to postural control [79]. The brain receives and processes sensory input from the visual, vestibular, and somatosensory systems to maintain balance. Cognitive decline affects the ability of the brain to accurately integrate and interpret these sensory signals, leading to difficulty maintaining balance and spatial orientation. Cognitive function involves planning and performing complex motor tasks including walking and changing direction [80]. With cognitive decline, there could be disturbances in gait patterns, such as shortened stride length, altered timing, or less efficient weight transfer, all of which affect balance.

Maintaining balance requires concentration, especially in challenging situations or when navigating complex environments. Cognitive decline could lead to decreased concentration and greater susceptibility to distractions, making it harder to maintain balance and avoid danger [81]. Cognitive decline slows down reactions, affecting the ability to respond quickly to sudden changes or disturbances that challenge balance. Slower reaction times could lead to difficulty recovering from slips, trips, or unexpected movements, increasing the risk of falls. Simultaneous performance of cognitive and motor tasks results in dual-task interference, in which one or both tasks are affected.

### 2.6. Interaction between cognitive function and motor control

As people age, the interplay between cognitive function and motor control becomes more pronounced. Cognitive function refers to mental processes, while motor control involves the ability to coordinate movement and perform physical activities. Cognitive function and motor control undergo various changes as we aging [82]. Older adults experience changes in attention, such as a decreased ability to multitask or difficulty filtering out irrelevant information. These changes in attention could affect motor performance, leading to reduced accuracy and slower reaction times in complex motor tasks. Dual tasking involves performing two tasks at the same time. As people age, the ability to manage dual tasks could suffer. Performing motor tasks alongside cognitive activities could become more challenging. This dual-task interference could lead to impaired motor control and increased risk of falls in older adults.

Memory plays a vital role in motor learning and skill retention. Age-related changes in memory processes affect motor skill acquisition and retention. Older adults need more practice to learn new motor tasks and recall of previously learned motor skills may also be affected. Cognitive functions related to spatial awareness and navigation are important for motor control. Changes in these cognitive processes could affect the ability of an individual to navigate in an environment, leading to difficulty maintaining balance and avoiding obstacles. Cognitive functions related to emotional processing could influence motor control. Anxiety or fear of falling, for example, could affect an individual's confidence in their ability to exercise, leading to more cautious and restricted movement. As we aging, brain regions responsible for cognitive processing and motor control could experience structural and functional changes that affect their interactions [83]. Older adults may develop compensatory strategies to manage cognitive and motor changes. For example, they could rely on mastered motor skills or use external cues to aid performance. Understanding the interplay between cognitive function and motor control during aging is critical to promoting healthy aging and maintaining functional independence [84]. Balance training exercises that challenge cognitive aspects can help improve motor and cognitive performance. A comprehensive approach addressing cognitive and motor aspects would promote healthy aging.

### 2.7. The role of underlying neural mechanisms in age-related balance disorders

Various underlying neural mechanisms, involving changes in the brain and nervous system, influence age-related decreases in balance [85, 86]. As people age, certain structural and functional changes occur in the neural pathways responsible for balance control. Neurotransmitters are chemical messengers that facilitate communication between neural cells. In age-related decrease in balance, levels of neurotransmitters that play a crucial role in motor control and balance regulation may change [63]. Changes in neurotransmitter function could disrupt motor coordination and lead to balance problems. Certain brain regions involved in balance control such as the cerebellum, basal ganglia, and vestibular system could involve age-related degeneration.

As mentioned before, cerebellum is responsible for coordinating movement and balance, while the basal ganglia play a role in regulating muscle tone and motor initiation [87]. Degeneration of these areas could impair motor coordination and balance. White matter in the brain is made up of nerve fibers that form connections between different brain regions. Age-related white matter changes, such as myelin degeneration, could lead to slower neural signaling and reduced communication between brain regions involved in balance control [88]. Brain volume gradually decreases with age, including areas associated with motor control and balance. Loss of brain volume affects the efficiency of neural processing and leads to age-related declines in balance. Neuroplasticity plays a significant role in age-related decline in balance. When changes occur in certain brain regions, the brain may try to compensate for these changes by reorganizing neural connections or relying on alternative balance control strategies. However, the degree of compensation varies from person to person and does not fully offset age-related declines in balance.

Balance control involves integrating sensory input from the visual, vestibular, and somatosensory systems. Age-related changes in these sensory systems could affect sensorimotor integration, leading to difficulties in processing and interpreting sensory information to maintain balance. Age-related cognitive decline affects concentration, motor planning, and dual-task performance, all of which contribute to balance disturbances. The interplay of these neural mechanisms contributes to age-related decline in balance, leading to increased fall risk and restricted mobility. It is important to note, however, that age-related changes in balance are not inevitable and could be influenced by lifestyle factors, physical activity, and interventions aimed at promoting brain health and balance.

## 3. Aging-related changes in the musculoskeletal system

### 3.1. Effects of muscle weakness on balance

Muscle weakness can have a major impact on balance, as muscles play a vital role in providing stability and support during a variety of movements and activities [89]. The ability to maintain balance relies on the interaction between the sensory and muscular systems. Weak or underpowered muscles cause balance problems and increase the risk of falls. Muscles are responsible for maintaining posture and stability during standing, walking, and running activities. Weak muscles make it difficult to provide the support necessary to maintain an upright posture, resulting in a stooped or swaying posture, which increases the risk of falling [90]. Leg muscle weakness can lead to changes in gait patterns such as shuffling, shorter, or uneven stride lengths. This gait abnormality disrupts normal walking patterns and impairs balance. Weight shifting is an important part of balance control [23]. Muscle weakness hinders effective weight transfer from one leg to the other, resulting in instability when walking or turning. Strong muscles are essential for quick, coordinated responses to unexpected perturbations or balance challenges. Muscles function as stabilizers for joints, helping to keep them in proper alignment during movement. Muscle weakness could not stabilize the joint sufficiently, causing joint instability and increasing the risk of falls [91]. Proprioception relies on feedback from muscles and joints to provide information about body position and movement. Muscle weakness impairs proprioceptive feedback, making it harder for the brain to accurately perceive where the body is in space. Muscle weakness leads to fatigue more quickly, making it more difficult to maintain balance, increasing the risk of falls.

### 3.2. Effects of reduced joint mobility on balance

Decreased joint mobility has a major impact on stability, especially when maintaining balance and performing a variety of physical activities [91]. Joint mobility refers to the joint's ability to move freely without causing pain or discomfort. Over time, age, physical inactivity, and certain health conditions could lead to decreased joint mobility. Reduced joint mobility, limiting the joint's full range of motion, affects balance and stability. During activities such as walking or reaching for objects, the human body needs to transfer its weight from one leg to the other or from one side to the other. Lack of mobility in the hips, knees, or ankles could reduce the efficiency of shifting weight, causing instability, and increasing the risk of falls. Reduced joint mobility could lead to changes in gait patterns that impair stability during walking and increase the likelihood of stumbling or tripping. Reduced joint mobility impedes the body's ability to adjust quickly when encountering an unexpected obstacle, resulting in a fall. Joint mobility plays a role in maintaining proper postural alignment. Stiffness and lack of mobility in certain joints could lead to poor posture, which affects balance and stability [92]. Difficulty performing these tasks could compromise overall stability and increase the risk of falls.

### 3.3. Effects of changes in bone density on balance

Loss of bone mass in osteoporosis, common in older adults, could significantly lead to balance problems and increase the risk of falls [93]. Osteoporosis is a disease characterized by weakened bones. The impact of osteoporosis on balance is of particular concern for older adults, as falls have profound consequences, leading to injury and reduced mobility. Over time, osteoporosis causes bones to lose density and strength. Weak bones are more likely to break even from minor shocks or during everyday activities [94]. Changes in bone structure and density in osteoporosis could affect posture. The spine could become compressed or fractured, leading to a hunched or hunched posture, a change in posture that upsets the body's center of gravity and impairs balance.

In osteoporosis, weakened bones provide less support during weight-bearing activities like exercise, such as walking or climbing stairs, causing instability, and affecting balance control during these activities. Osteoporosis-related fractures in weight-bearing joints, such as the hip or knee, could cause severe pain, impaired mobility, and prolonged recovery time [95]. These factors increase the risk of falls because people could be hesitant to move or engage in physical activity for fear of injury. Osteoporosis could alter gait patterns, changes that disrupt natural walking patterns and affect balance control. Osteoporosis-related fractures or joint changes could affect the feedback provided by the proprioceptive system, which relies on information from bones, muscles, and joints to sense body position [96]. Reduced proprioceptive feedback makes it difficult to adjust body position for balance.

### 3.4. The key role of the pelvic region for balance

The pelvic area is an important part of the human body and serves several functions. It is the complex bony structure at the base of the spine that plays a fundamental role in supporting and connecting the upper body [97]. The pelvis provides a stable base for the spine is a strong platform for the attachment of various muscles and tendons and helps maintain an upright posture and balance during standing, walking, and other activities [98]. The pelvis is critical for many types of movement including walking, running, bending, and sitting. It acts as a pivot point for lower body and trunk movement, facilitating efficient locomotion and body movement [99]. The pelvis makes a major contribution to maintaining the body's center of gravity and distributing weight evenly between the two lower extremities. It helps maintain balance and stability while standing and moving. Overall, the pelvic region serves a variety of functions and is critical to all aspects of human anatomy and physiology.

## 4. Genetics, inflammation and autoimmunity are out of balance with aging

### 4.1. Genetic factors and imbalance in the elderly

Genetic factors could also play a role in the development of balance disorders in older adults [100]. While aging itself is a risk factor for balance problems, certain genetic variations and mutations further increase susceptibility to balance-related problems. Variations in genes involved in the development and function of the vestibular phylogeny affect balance and stability. For example, mutations in genes encoding proteins responsible for maintaining the structure of the vestibular system. The integrity of the inner ear or its ability to transmit vestibular signals to the brain could lead to vestibular dysfunction and balance disturbances. Several genetic factors increase the risk of neurodegenerative diseases. Gene mutations associated with inner ear disorders could disrupt the normal function of the inner ear and cause balance problems.

Genetic factors affect an individual's susceptibility to vascular problems, such as blood vessel-related balance disorders, which can occur when blood flow to the brain or inner ear is compromised after a stroke or acute cerebrovascular disease. Genetic factors could influence an individual's musculoskeletal health, which affects overall body stability and balance. Genetic variants affecting neuroplasticity affect how the brain compensates for age-related changes in the vestibular and balance systems. While genetic factors increase the risk of balance disorders in older adults, they often interact with other environmental and lifestyle factors. Aging, environment, medical conditions, and lifestyle choices could all affect the expression of genetic traits and the likelihood of balance problems [101].

### 4.2. Inflammatory and autoimmune diseases and imbalance in the elderly

Inflammatory and autoimmune diseases also throw off balance in older adults [102, 103]. Inflammation is the body's natural response to injury or infection, but when it becomes chronic or dysregulated, it causes damage to various tissues including the nervous system. Autoimmune diseases occur when the immune system mistakenly attacks its own healthy cells and tissues. Both inflammatory and autoimmune processes affect the central nervous system, including structures and pathways responsible for balance and motor control. Vestibular neuritis is a disease characterized by inflammation of the vestibular nerve that connects the inner ear to the brainstem. This inflammation disrupts the transmission of balance-related signals from the inner ear to the brain, leading to symptoms such as vertigo and lightheadedness. Autoimmune inner ear disease (AIED) is an autoimmune disorder, in which the immune system's attack on these structures could lead to sensorineural hearing loss and balance problems [104]. Multiple sclerosis is an autoimmune disease that affects the CNS, and in some cases, inflammation and demyelination could affect the vestibular system, causing balance disturbances and vertigo [105]. Rheumatoid arthritis (RA) is a chronic autoimmune disease that could also lead to systemic inflammation [106]. Inflammation of the inner ear or nervous system could cause balance problems in some people with rheumatoid arthritis. Systemic lupus erythematosus (SLE) is a systemic autoimmune disease in which nervous system involvement could lead to a variety of symptoms, including balance and gait disturbances [107]. Giant cell arteritis (GCA) is an inflammatory disease that primarily affects blood vessels, and inflammation of these blood vessels leads to balance problems and vision disturbances. Sarcoidosis is an inflammatory disease that affects the CNS, resulting in a variety of neurological symptoms. Taken together, inflammatory and autoimmune responses could directly damage structures involved in balance and motor control, disrupt neural pathways, or lead to other systemic effects that indirectly affect balance. Early detection and intervention are critical to mitigate the impact of these conditions on the balance of the aging population.

## 5. Early warning signs of balance disorder

### 5.1. Early signs of common balance problems

Recognizing early signs of balance problems is very important for timely intervention and prevention of falls and injuries. The most common early sign of balance problems is a constant feeling of instability, even when standing or walking on a flat surface [108]. This sensation could be described as feeling off balance or the ground is moving. Tripping over objects frequently during daily activities could indicate an underlying balance problem. Difficulty maintaining an upright posture when standing, causing the body to slump or lean to one side, could be an early sign of a balance problem [109]. Experiencing a fall for no apparent reason is a major red flag for a balance problem. If falls occur in situations that previously did not cause a problem or become frequent, they should be paid attention. Balance problems when turning, reaching, or bending indicate a problem with the vestibular or proprioceptive system.

Feelings of dizziness or vertigo related to the inner ear or other vestibular problems could affect balance. A marked decrease in coordination, resulting in difficulty with fine motor tasks or overall clumsiness, could indicate an underlying balance problem. Even in the absence of immediate danger, an excessive fear of falling was a psychological response to early balance problems. Struggling to perform two tasks at once, such as walking and talking or carrying objects while moving, could indicate an underlying balance problem [110]. Difficulty judging distance or navigating in familiar environments were related to problems with spatial awareness and balance [111]. Changes in walking patterns such as shuffling, shortening your stride, or walking slower than usual, could also be early signs of balance problems. Feeling more fatigued than usual or physically tired when performing previously fewer demanding activities indicates a balance problem. Be aware that some of these symptoms were caused by other medical conditions or medications. Therefore, a comprehensive evaluation by a doctor or physical therapist is essential to accurately identify the root cause of balance problems.

### 5.2. Assessing postural stability in older adults

Assessing postural stability in older adults involves a variety of methods and tests that help assess their balance and identify potential balance impairments. The Berg Balance Scale (BBS) is a widely used assessment tool to measure an ability to *balance* [112]. It consists of a series of 14 functional tasks that assess static and dynamic balance, such as standing with feet together, turning and extending. Each task is scored based on an individual's ability to complete the task, with a maximum score of 56 indicating good balance. The timed up and go test (TUG) measures the time it takes for a person to stand up from a chair, walk three meters, turn around, walk back to the chair, and sit down [19]. It assesses dynamic balance, gait, and functional mobility. Longer TUG times indicate balance and mobility problems. This test assesses an ability to maintain balance while standing on one leg for a specified period (usually 30 seconds). It assesses single-leg stability and weight-bearing capacity. Functional stretch test (FST) measures an ability to reach forward while maintaining a stable base of support. It assesses homeostasis and the ability to recover from forward stretching movements [113]. This test assesses the ability to straddle four canes placed in a square pattern on the floor, testing dynamic balance, coordination, and agility. Sensory organization test (SOT) assesses an ability to maintain balance using visual, somatosensory, and vestibular inputs [114]. It is performed using a computer dynamic posturography system and provides valuable information on the contribution of different sensory systems to postural stability. Observing an individual's gait could provide insight into their balance and mobility. Gait analysis includes assessment of stride length, stride width, cadence, and any abnormalities or asymmetries in the walking pattern. Various functional mobility tests such as walking on an uneven surface, stepping over an obstacle, or performing a tandem walk, help assess the ability of individuals to maintain balance during daily activity. A balance confidence scale (ABC) assesses an individual self-perceived confidence in performing a specific activity without falling [115]. These scales provide valuable information on the psychological aspects of balance control. A thorough evaluation of the medical history and physical examination help identify underlying medical conditions or medications that was causing balance problems [116]. These assessments should be performed in a safe environment with appropriate supervision to prevent falls during testing.

### 5.3. Abnormal gait as a potential indicator of balance problems

Identifying abnormalities in gait could serve as a potential indicator of balance problems [117]. Gait abnormalities are deviations or changes in a person's walking pattern. Significant differences in stride length between the left and right legs may indicate an asymmetry in strength or motor control, which affects balance. A sluggish gait, in which the feet barely leave the ground when walking, could indicate muscle weakness or impaired coordination that could lead to balance problems [118]. Decreased arm swing while walking indicates difficulty coordinating upper body movements, which affect balance and stability. An unusually large distance between your feet while walking suggests trying to increase stability for balance reasons. Binge gait, which is walking with small, fast steps, is common in PD that affects balance and coordination. Walking on tiptoe or shuffling due to difficulty lifting the foot indicate muscle weakness or a neurological problem affecting balance [119]. In Trendelenburg gait, the pelvis tilts excessively to one side during walking, indicating hip abductor muscle weakness [120]. Excessive side-to-side rocking during walking could be a sign of a balance problem, especially when a person struggles to maintain a straight path. If the person has difficulty starting to walk or stops suddenly, indicating a problem with motor planning or coordination that affects balance. Hip rocking from side to side during walking usually indicates muscle weakness or joint problems that affect balance [121]. Significant differences in left and right arm swing indicate an imbalance in muscle strength or coordination, which affects overall balance. Sudden or noticeable changes in walking speed that may be related to balance disorders or other health problems.

### 5.4. Mechanisms of early balance disorder formation

Early onset of balance disorders could be attributed to age-related changes, medical conditions, and lifestyle factors [122]. The mechanisms underlying these deficits are complex and vary individually. Physiological changes occur in the musculoskeletal, sensory, and nervous systems as individuals age [123]. Muscle mass and strength could decrease, affecting the ability to generate enough force to maintain balance. Changes in proprioception and vestibular function also lead to balance deficits. Age-related changes in the brain and nervous system could affect balance control [124]. Structural and functional changes in the cerebellum and basal ganglia involved in balance affect motor coordination and postural control [125]. Certain medicines could cause dizziness, drowsiness, or changes in balance that increase the risk of falling. Various medical conditions could cause early balance deficits. Includes inner ear disorders, neurological disorders, orthopedic problems, and cardiovascular disease.

Age-related cognitive changes affect attention, reaction time, and executive function, making it more challenging to process sensory information and adapt to balance challenges. Generalized muscle weakness, often associated with a sedentary lifestyle, can impair postural stability, and increase the risk of falls. Balance requires the integration of sensory information from the visual, vestibular, and somatosensory systems; any damage in these systems could lead to a balance deficit [126]. A sedentary lifestyle can lead to muscle weakness, decreased proprioceptive awareness, and reduced confidence in balance control. Clutter, uneven surfaces, or poor lighting in the living environment increase the risk of tripping and falling, especially for people with early balance deficits. Anxiety or fear of falling can lead to altered gait patterns and decreased physical activity, further exacerbating balance deficits.

Understanding the underlying mechanisms of early balance deficits could guide interventions aimed at improving balance and preventing falls. Early detection and appropriate implementation of balance training exercises, strength training, and environmental modification are critical to minimizing the effects of balance deficits and improving overall mobility and quality of life [127]. In the event of early balance deficits, it is advisable to seek evaluation and guidance from a physician or physiotherapist for appropriate assessment and individualized intervention strategies.

## 6. Prevention and intervention strategies

### 6.1. The importance of early detection and intervention of balance problems

Early detection and intervention of balance problems is critical as they can significantly impact an individual’s health, well-being, and overall quality of life. Balance problems increase the risk of falls, which lead to serious injuries, broken bones, hospitalization, and long-term disability. Falls and balance problems could seriously affect individual confidence and mental health. Early intervention helps individuals maintain self-esteem and reduce anxiety associated with mobility problems. Falls caused by balance problems lead to significant medical costs in hospitalization, rehabilitation, and long-term care. Early intervention helps prevent falls and associated medical costs. Maintaining a good balance is essential to maintaining mobility and independence in everyday activities. Addressing balance issues early on help individuals maintain an active and engaged lifestyle, resulting in a better quality of life.

Improved balance allows people to participate in social activities, exercise, and hobbies without fear of falling or being restricted. Balance problems sometimes be a symptom of an underlying medical condition such as a vestibular disorder, neurological disorder, or musculoskeletal problem. Early detection allows healthcare professionals to identify and address these conditions, improving overall health outcomes. Balance problems could lead to secondary complications such as muscle weakness, reduced activity levels, and social isolation. Early intervention could prevent or minimize these complications. Additionally, early detection of balance problems enables individuals and caregivers to make appropriate modifications to the living environment to reduce fall risk and improve safety, promoting healthy aging. Overall, early detection and intervention of balance problems has many benefits, including preventing falls, improving mobility, improving quality of life, reducing healthcare costs, and identifying and treating underlying conditions [128]. Timely assessment and individualized intervention could significantly improve the balance, safety, and overall well-being of individuals experiencing balance problems.

### 6.2. Exercises to improve balance and stability in older adults

Exercise aimed at improving balance and stability in older adults significantly enhances their overall mobility and reduce the risk of falls [129]. These programs typically focus on strengthening key muscle groups, enhancing proprioception and sensory integration, and practicing specific balance exercises [130]. A medical professional or qualified exercise specialist must be consulted before beginning any new exercise program. Effective exercises for improving balance and stability in older adults include: 1) Tai Chi: tai chi is a low-impact mind-body exercise that emphasizes slow, fluid movements and shifting of weight. It promotes balance, coordination and flexibility while reducing stress [131]. 2) Yoga: yoga consists of a variety of poses and poses that challenge balance and flexibility. It can help improve core strength and stability while promoting relaxation and body awareness [132]. 3) Standing on one leg: stand near a sturdy support and lift one foot off the ground. Hold this position for 10-30 seconds, then switch legs [133]. Gradually the duration increases as balance improves. 4) Heel-to-toe walk: walk in a straight line, placing the heel of one foot directly in front of the toes of the other foot. This exercise challenges balance and coordination [134]. 5) Leg lifts: stand behind a sturdy chair for support. Lift one leg straight out to the side, then return to the original position. Repeat on the other leg. This exercise targets the hip abductors [135]. 6) Tandem stand: stand with one foot in front of the other (heel-to-toe position) and try to hold this position for 20-30 seconds. Switch foot positions and repeat [136]. 7) Sit-stand exercise: Practice standing up from a chair without using your hands for support. This exercise strengthens the leg muscles and improves balance during transitional movements. 8) Walk in place: lift one knee toward your chest, then lower and lift the other knee [137]. Repeat this marching motion for 30 seconds to 1 minute. 9) Toss: stand facing your partner and gently toss a soft ball back and forth. The action of catching and throwing the ball challenges balance and reaction time. 10) Balance exercises: use a balance board or wobble board to challenge and improve balance control [138]. Start with both feet on the board and gradually transition to a single-leg position as your balance improves. 11) Resistance training: includes strength exercises that target major muscle groups, such as squats, lungs, and calf raises [139]. Strengthening these muscles provides stability during sports and everyday activities. 12) Ankle circles and toe taps: perform ankle circles to improve ankle mobility and flexibility [140]. Also, practice tapping your toes while sitting to strengthen the muscles in your feet and ankles.

Remember to start with exercises that are appropriate for your current fitness level and gradually increase the difficulty and intensity. Perform the exercise on a stable surface first, then gradually progress to unstable surfaces as your balance improves. Aim to incorporate balanced exercises into your routine at least two to three times a week. Consistency is key to achieving positive results over time. It is recommended to work with a physical therapist or exercise specialist to customize an exercise program to meet your specific needs, address any existing medical conditions, and ensure safe and effective improvement of balance and stability.

### 6.3. Fall prevention strategies and their effectiveness

The effectiveness of a fall prevention strategy depends on multiple factors, including individual overall health, adherence to the strategy, and the presence of specific risk factors. Multifactorial interventions that combine multiple fall prevention strategies are more effective in reducing fall rates than single interventions.

### 6.4. Impact of interventions on balancing mechanisms

Interventions aimed at improving balance in older adults work through various mechanisms of action, targeting various aspects of balance control and related physiological processes. The impact of these interventions on balance mechanisms can be substantial, resulting in improved postural stability and reduced risk of falls. The first is muscle strength and coordination training involving balance and gait control [142]. By increasing muscular strength and endurance, individuals could better support body weight and maintain stability during activity. Balance exercises challenge neuromuscular coordination and require the integration of sensory input and motor responses. Practicing coordinated movements improves the efficiency with which the nervous system controls balance. This is followed by enhanced proprioceptive balance and sensory integration training, which help individuals become more aware of the position of their body in space and improve the accuracy of postural adjustments. The vestibular system is responsible for the perception of motion and spatial orientation. Vestibular exercises help improve the brain’s ability to process vestibular input, resulting in better balance control. Balance interventions typically involve integrating sensory input from the visual, vestibular, and somatosensory systems [143]. By training the brain to process and prioritize sensory information, individuals could maintain better balance in different environments. Finally, balance exercises teach individuals various postural control strategies such as weight shifting, stride strategy and anticipation adjustments, dual-tasking exercises, and confidence-boosting and fear-reducing exercises, which enhance the ability to recover from disturbances and prevent falls [144].

Regular practice and consistency are key to maximizing training benefits and maintaining gains achieved through balanced interventions. As a result, older adults could improve balance, reduce the risk of falls, increase independence, and increase confidence in daily activities.

## 7. Clinical Assessment Methods for Balance Changes

### 7.1. Overview of commonly used balance assessment methods

Various balance assessment tools are used to assess an individual's balance ability, identify potential balance deficits, and track progress during intervention. Here is an overview of some commonly used balance assessment tools:

Berg Balance Scale (BBS) [145]*:* BBS is a widely used assessment tool to assess the balance ability of the elderly and individuals with balance disorders. It consists of 14 functional tasks with a maximum score of 56 indicating good balance. Timed Up and Go Test (TUG) [146]: TUG measures the time it takes for an individual to stand up from a chair, walk three meters, turn around, walk back to the chair, and sit down again. Dynamic Gait Index (DGI) [147]: DGI assesses an individual's ability to adjust their gait to meet different tasks and environmental challenges. It consists of eight tasks. Functional Reach Test (FRT) [148]: The FRT measures an individual's ability to reach forward while maintaining a stable base of support. Single Leg Standing Test (SLST) [149]*:* TLST assesses an individual's ability to maintain balance while standing on one leg for a specified amount of time (e.g., 30 seconds). Four-Square Step Test [150]: The Four-Square Step Test assesses an individual's ability to straddle four square canes placed on the floor, testing dynamic balance, coordination, and agility. Sensory Organization Test (SOT) [151]: SOT uses a computerized ambulatory postography system to assess an individual's ability to maintain balance using visual, vestibular, and somatosensory inputs. It provides information on the contribution of different sensory systems to postural stability. Activity-specific Balance Confidence Scale (ABC) [152]: ABC assesses an individual's self-perceived confidence in performing a specific activity without falling. Mini-Balance Evaluation Systems Test (Mini-BESTest) [71]: Mini-BESTest assesses various components of balance, including anticipatory postural adjustment, reactive postural control, and sensory orientation. Tinetti Performance-Oriented Mobility Assessment (POMA) [153]: POMA assesses the individual's gait and balance during various functional tasks and provides indicators that reflect the performance of balance and mobility. overall score.

These assessment tools were used alone or in combination, depending on the specific needs of the individual and the clinical or research context. They provide invaluable information for designing individualized balance training programs, tracking progress, and identifying individuals at risk for falls or balance-related problems. Additionally, these assessments help healthcare professionals make informed decisions about appropriate interventions and fall prevention strategies.

### 7.2. Balancing the validity and reliability of the assessment method

The validity and reliability of balance assessment methods are important considerations in evaluating the validity and accuracy of these tools in measuring an individual's balance ability. Validity refers to the degree to which an assessment tool measures what it is intended to measure, while reliability indicates the consistency and stability of the results produced by an assessment method. High validity and reliability are essential to ensure that balance assessments provide accurate and consistent information about an individual's balance state.

Validity includes structure, content, and standard validity. Structural validity evaluates whether the detection tool accurately measures the expected structure. Content validity determines whether the items or tasks included in the assessment tool adequately represent the full range of balance-related competencies that the tool is designed to measure. The standard effectiveness assessment assesses whether the results are consistent with those obtained from a recognized “gold standard” assessment or established balancing measures. Repeated reliability assessment assesses the consistency of results when an instrument assesses the same individual on two separate occasions. High repeatability indicates that the assessment produces consistent results over time. Inter-rater reliability assesses the consistency of assessment results when different raters or reviewers use the tool on the same individual. High inter-rate reliability indicates that the assessment was not influenced by the subjective judgment of the reviewers. Internal consistency reliability measures the consistency of items or tasks within an assessment tool. It assesses whether all items in an assessment measure the same structure and provide consistent results. Sensitivity and specificity are additional important considerations when assessing the validity of a balance assessment tool [154]. Sensitivity refers to the ability of an assessment tool to accurately identify individuals with balance deficits or who are at risk of falls. Specificity represents the ability of the assessment tool to correctly identify individuals with no balance deficit or risk of falls. To determine validity and reliability, assessment tools are often tested in studies with different populations. Psychometric properties such as correlation coefficient, intraclass correlation coefficient (ICC), and Cronbach's alpha are used to quantify the validity and reliability of the assessment method [155].

### 7.3. Application of integration techniques for objective balance assessment

In recent years, ensemble techniques for objective balance assessments have become increasingly popularity because of their multiple advantages over traditional assessment methods. Technology-based balance assessment tools provide objective, quantitative data, improve accuracy and reliability, allow real-time feedback, and facilitate remote monitoring. The use of technology for objective balance assessments has developed rapidly, and the more commonly used ones are:

1. Force plates and pressure sensors [156]*:* force plates and pressure sensors are used to measure the force exerted by the individual feet during standing or dynamic movement. power equipment. By analyzing pressure distribution and pressure swing centers, these tools provide detailed information about an individual's postural stability and balance control.
2. Inertial measurement units (IMU) [157]*:* IMU consist of accelerometers, gyroscopes, and magnetometers to measure the orientation and motion of the body in space. IMUs can be placed on various parts of the body to assess dynamic balance during walking, turning, or other functional tasks.
3. Wearable sensors [158]*:* accelerometers or gyroscopes in wearable sensor devices can continuously monitor an individual's movement and provide data on daily activities, gait patterns, and posture changes.
4. Computerized dynamic posturography (CDP) [159]*:* CDP systems use force plates or rocking reference platforms to assess an individual's postural responses to different sensory conditions and challenges. These systems could identify specific balance disorders and aid in the development of individualized intervention plans.
5. Virtual reality (VR) and augmented reality (AR) [160, 161]*:* VR and AR technologies are increasingly used in balance assessments to create immersive environments that challenge individual balance and motor responses.
6. Mobile application [162]*:* There are various mobile applications (Apps) designed for balance assessment and training. These apps typically use built-in sensors in a smartphone or tablet to measure postural sway and provide interactive exercises to improve balance.
7. Video-based analysis (VBA) [163]*:* VBA software was used to record and analyze an individual movement during balance tasks, providing valuable visual feedback and allowing detailed assessment of gait and postural control.
8. Telehealth and remote monitoring [164]*:* technology could enable remote balance assessment and monitoring, which is especially useful for individuals who could not easily travel to a clinical setting. Remote balance assessments were performed via video conferencing or using wearable devices that transmit data to healthcare providers.

Integrating technology into balance assessments allows for more precise and comprehensive data collection, personalized interventions, and objective monitoring of progress over time.

### 7.4. Mechanism and clinical significance of balanced assessment techniques

Balance assessment techniques use various mechanisms to assess an individual balance ability and identify potential balance deficits. These mechanisms are based on principles of biomechanics, neuromuscular control, and sensory integration. Understanding the mechanisms of these assessment techniques is critical to interpreting results and determining their clinical significance. Some common mechanisms and their clinical implications in balance assessment include:

1. Postural sway analysis measures involuntary movements of the body's center of pressure (CoP) during standing [165]. It assesses the body’s ability to maintain stability while maintaining an upright posture. The clinical significance is to identify subtle balance impairments and early signs of balance instability, helping to distinguish people with normal balance from those at risk of falls.
2. Sensory organization tests assess an individual's ability to use visual, proprioceptive, and vestibular inputs to maintain balance [114]. Its clinical significance is to help identify sensory deficits and assist in planning targeted interventions based on specific sensory challenges.
3. Gait analysis evaluates an individual walking pattern and assesses the dynamic balance and efficiency of the walking pattern [166]. Its clinical implications could guide interventions that improve walking stability and reduce the risk of falls.
4. Functional stretch tests the individual maximum reach while maintaining a stable support base [167]. Its clinical significance is to provide valuable information about an individual homeostasis and the risk of falls.
5. The timed priming test assesses an individual functional mobility by measuring the time it takes to stand up from a chair, walk three meters, turn around, walk back, and sit down [168]. Its clinical significance is to assess fall risk and functional mobility. It helps identify individuals who may benefit from balance training and fall prevention interventions.
6. The Single leg standing test assesses an individual ability to maintain balance while standing on one leg [169]. The clinical significance is to help identify individuals with balance deficits or lower extremity weakness so that targeted interventions were implemented to improve single-leg stability.
7. Four square step test assesses an individual ability to straddle four canes in a square pattern placed on the floor [150]. Its clinical implications could guide interventions to improve quick strides and reduce the risk of falls during complex movements.

The clinical significance of these balance assessment techniques lies in their ability to identify balance deficits in different clinical populations, guide intervention planning, track progress, and assess fall risk. By understanding the mechanisms behind these techniques, medical professionals could make informed decisions regarding the appropriate use of balance assessment tools and the development of tailored balance rehabilitation programs for patients with balance disorders.

## 8. Address comorbidities and drug therapy

### 8.1. Effects of age-related health conditions on balance

Various physical and sensory changes occur as individuals age, and age-related health conditions have a major impact on balance. These changes affect musculoskeletal, sensory, and nervous system functions involved in maintaining balance. Some common age-related health conditions and their effects on balance include muscle weakness and atrophy, decreased proprioception, vestibular disorders, visual changes, neurological disorders, orthopedic problems, cardiovascular disease, peripheral neuropathy, cognitive abilities decline, and drug side effects. The combination of these age-related health conditions could lead to increased instability, increased risk of falls, and decreased overall mobility. Falls in older adults could have profound consequences, including broken bones, hospitalization, and loss of independence. Therefore, it is critical to proactively address these health conditions, regularly assess balance, and implement appropriate interventions to improve balance, prevent falls, and improve the overall quality of life of older adults. Balance training, strength exercises, home improvements, and medication management are some strategies that can lessen the impact of age-related health conditions on balance.

### 8.2. Drugs and Alternatives That may Cause Balance

Certain medicines could cause balance problems, dizziness, or impaired coordination, especially in older adults. It is critical for healthcare providers to be aware of these potential side effects and consider alternative medications when appropriate. The following are some drug classes known to affect balance, along with potential alternatives:

1. Benzodiazepines are used to treat anxiety and sleep disturbances, but may cause drowsiness, dizziness, and impaired balance.
2. Antidepressants, especially tricyclic antidepressants (TCAs) and certain selective serotonin reuptake inhibitors (SSRIs), could cause dizziness and balance disturbances.
3. Certain antipsychotics, especially first-generation antipsychotics, may cause sedation and balance problems.
4. Antihistamines used to treat allergies may cause drowsiness and affect balance.
5. Certain antiepileptic drugs such as phenytoin and carbamazepine, may cause dizziness and imbalance.
6. Alpha-blockers used to treat hypertension may cause orthostatic hypotension and increase the risk of falls.
7. Diuretics could cause electrolyte imbalances, leading to dizziness and dehydration.

It is worth noting that drug changes should be conducted under the guidance and supervision of professional medical personnel. In some cases, the benefits of a particular drug may outweigh the risks of balance problems, especially if suitable alternatives are unavailable. An individual medical history, current health condition, and medication regimen should be carefully evaluated before making any changes to medications. For older adults experiencing balance problems, a comprehensive evaluation including a medication review can help identify underlying factors for balance problems and determine the most appropriate management plan.

### 8.3. Mechanisms underlying interactions between aging/drugs and homeostasis

The interplay between comorbidities, medications, and balance in older adults can be complex and multifactorial. There are several mechanisms that influence how these factors interact and affect an individual's balance:

1. Comorbidities such as cardiovascular disease, diabetes or musculoskeletal disease in old age induced physiological changes in the body. These changes could affect muscle strength, joint mobility, sensory function, and overall physical fitness.
2. Many drugs used to treat diseases of aging could have side effects that directly or indirectly affect balance. Common side effects include dizziness, drowsiness, orthostatic hypotension, and disturbances in coordination and reaction time.
3. Age-related vision and hearing loss, which could affect an individual's sensory input.
4. Elderly people with multiple comorbidities often take multiple drugs at the same time. Drug combinations may interact and exacerbate side effects, increase balance problems and risk of falls.
5. Some medications could interact, causing unintended side effects and affecting balance.
6. Medications and comorbidities that affect cognition, attention, and executive function could affect an individual's ability to process sensory information and make appropriate postural adjustments to maintain balance.
7. Geriatric comorbidities and medications could lead to nutritional deficiencies, which are associated with muscle weakness and balance disorders.
8. Medications and comorbidities could affect the vestibular and proprioceptive systems, which are critical for balance and spatial orientation.

Managing the interplay between geriatric comorbidities/drugs and balance requires a holistic and individualized approach. Healthcare providers must carefully evaluate an individual’s medical history, medication regimen, and balance ability. Management plans may include optimizing treatment of geriatric comorbidities, adjusting medications to minimize side effects, providing balance training and fall prevention strategies, addressing sensory impairments, and promoting overall health and well-being.

## 9. Future directions for research and clinical practice

### 9.1. Promising areas of aging and homeostasis research

Research on aging and balance is an ongoing and dynamic field, and several promising areas merit further research to enhance our understanding and improve balance outcomes in older adults. Some areas include: studying the complex interplay and integration of sensory inputs such as vision, proprioception, and vestibular function in maintaining balance; individualizing balance training regimens according to specific impairments and risk factors; exploring technology-based interventions such as the efficacy of virtual reality, augmented reality, and wearable devices in improving balance and reducing fall risk in older adults; studying the complex interplay between cognitive functions such as attention, executive function, and memory, and motor control for maintaining balance; longitudinal evaluate the long-term effects of different balance training programs on fall rates, fall-related injuries, and overall quality of life in older adults; evaluate the effectiveness of home balance training programs in promoting adherence, convenience, and cost-effectiveness; develop and validate a comprehensive fall risk assessment tool and predictive models; investigate the impact of community-based falls prevention programs and initiatives involving collaboration between health workers, community-based organizations and public health agencies; Examine the effectiveness of joint interventions that simultaneously address multiple aspects of balance impairment; explore the relationship between frailty and balance in older adults; and understand how addressing frailty could positively impact balance and fall risk. Such research has the potential to inform evidence-based interventions and strategies for preventing falls, enhancing balance, and promoting healthy aging in older adults. By identifying effective approaches, researchers can facilitate the development of targeted and comprehensive balanced interventions to optimize functional independence and quality of life in the aging population.

### 9.2. Balancing assessment and innovation in intervention techniques

Several innovations in balance assessment and intervention techniques have emerged in recent years aimed at improving the accuracy, efficiency, and effectiveness of balance assessment and training. These innovations leverage technological and scientific advances to provide a more individualized and evidence-based approach to addressing balance among different populations. Some notable innovations include: wearable sensors and inertial measurement units, virtual and augmented reality, gamified balance training, telemedicine and remote monitoring, brain-computer interfaces (BCI), sports games and interactive training platforms, artificial intelligence and machines learning, haptic feedback devices, integration of comprehensive training methods, etc. [170-173]. These innovations provide a more precise and data-driven approach to advancing the field of balance assessment and intervention to address balance in older adults and those with neurological disorders. By utilizing technology and evidence-based practices, healthcare professionals could optimize balance training programs, reduce the risk of falls, and improve the overall mobility and quality of life of patients with balance disorders.

### 9.3. Advice for healthcare professionals

For healthcare professionals, a thorough, comprehensive assessment of the patient's balance abilities begins with a thorough, comprehensive assessment that includes identification of comorbidities, medication review, and assessment of sensory, musculoskeletal, and neurologic factors. Based on the results of the assessment, a personalized balance training program is developed for specific deficits and risk factors. Consider applying technology-based interventions and gamification training to improve engagement and adherence. This is followed by collaboration with other physical therapists, occupational therapists and pharmacists to provide a holistic approach to falls prevention and balance management [174]. Consider alternative regimens or dose adjustments as necessary. Finally, patients and their caregivers need to be educated about falls risks, home safety measures, and fall prevention strategies.

### 9.4. Understanding the mechanisms underlying age-related balance changes

Exploring future avenues of innovative research on the mechanisms underlying age-related balance changes and integrating multidisciplinary approaches will greatly advance the understanding of balance changes. Future research and directions include:

1. Neuroimaging studies such as fMRI, PET, and diffusion tensor imaging could provide insight into structural and functional changes in brain balance and motor control regions with age [175].
2. Adopting a systems biology approach could help to understand the complex interplay between genetic factors, epigenetics, cellular pathways, and environmental influences that lead to age-related changes in balance [176].
3. Conducting long-term longitudinal studies could capture the gradual nature of age-related balance changes and identify factors that contribute to accelerated or decelerated decline in balance [177].
4. Applying precision medicine principles to balance research could help identify individual risk profiles for balance disorders and guide personalized interventions based on genetic, physiological, and lifestyle factors [178].
5. The use of wearable sensors and big data analytics enables continuous monitoring of balance parameters in large populations, resulting in more robust datasets and a better understanding of age-related balance changes [179].
6. Studying the mechanisms of sensorimotor integration of balance control, particularly how sensory inputs are weighted and integrated, could provide insight into age-related changes in this process [180].
7. Further research into the role of cognitive functions such as attention, executive function, and memory in age-related changes in balance [82].
8. Consider the potential benefits of different types of exercise, such as resistance training, yoga, and tai chi, and explore optimal exercise and training modalities to mitigate age-related changes in balance [181].
9. Investigate the impact of social isolation, loneliness, and environmental factors on balance outcomes in older adults, and understand how social engagement and supportive environments affect balance [182]. These future research directions have the potential to deepen our understanding of the mechanisms underlying age-related balance changes and pave the way for more targeted and effective interventions to promote healthy aging, reduce the risk of falls, and improve the balance results in older adults. Integrating interdisciplinary collaboration and leveraging emerging technologies is critical to advancing our knowledge in the field.

## 10. Conclusion

The main findings of this review are: 1) Balance problems or dizziness are common among older adults. Disorders of vision, vestibular system, proprioception, and sensory integration are the key to age-related balance problems. 2) Muscle weakness, decreased joint mobility, changes in bone density, and neurodegenerative diseases, which could cause balance problems and falls. 3) Cognitive decline, side effects of drugs, comorbidity interactions could amplify fall risk and balance problems. Interaction between cognitive function and motor control affects balance in older adults. 4) In older adults, early detection of balance deficits is critical for timely intervention and fall prevention. During treatment, the integration of multimodal sensory input plays a vital role in maintaining balance in older adults. 5) Wearable sensors, VR, AR, and telemedicine are promising technologies for balance assessment and training. Advanced neuroimaging techniques provide insight into changes in brain structure and function associated with balance.
